# Early-Life Telomere Dynamics Differ between the Sexes and Predict Growth in the Barn Swallow (*Hirundo rustica*)

**DOI:** 10.1371/journal.pone.0142530

**Published:** 2015-11-13

**Authors:** Marco Parolini, Andrea Romano, Lela Khoriauli, Solomon G. Nergadze, Manuela Caprioli, Diego Rubolini, Marco Santagostino, Nicola Saino, Elena Giulotto

**Affiliations:** 1 Department of Biosciences, University of Milan, Milan, Italy; 2 Department of Biology and Biotechnology, University of Pavia, Pavia, Italy; Tulane University Health Sciences Center, UNITED STATES

## Abstract

Telomeres are conserved DNA-protein structures at the termini of eukaryotic chromosomes which contribute to maintenance of genome integrity, and their shortening leads to cell senescence, with negative consequences for organismal functions. Because telomere erosion is influenced by extrinsic and endogenous factors, telomere dynamics may provide a mechanistic basis for evolutionary and physiological trade-offs. Yet, knowledge of fundamental aspects of telomere biology under natural selection regimes, including sex- and context-dependent variation in early-life, and the covariation between telomere dynamics and growth, is scant. In this study of barn swallows (*Hirundo rustica*) we investigated the sex-dependent telomere erosion during nestling period, and the covariation between relative telomere length and body and plumage growth. Finally, we tested whether any covariation between growth traits and relative telomere length depends on the social environment, as influenced by sibling sex ratio. Relative telomere length declined on average over the period of nestling maximal growth rate (between 7 and 16 days of age) and differently covaried with initial relative telomere length in either sex. The frequency distribution of changes in relative telomere length was bimodal, with most nestlings decreasing and some increasing relative telomere length, but none of the offspring traits predicted the *a posteriori* identified group to which individual nestlings belonged. Tail and wing length increased with relative telomere length, but more steeply in males than females, and this relationship held both at the within- and among-broods levels. Moreover, the increase in plumage phenotypic values was steeper when the sex ratio of an individual’s siblings was female-biased. Our study provides evidence for telomere shortening during early life according to subtly different dynamics in either sex. Furthermore, it shows that the positive covariation between growth and relative telomere length depends on sex as well as social environment, in terms of sibling sex ratio.

## Introduction

Telomeres are nucleoproteins located at the termini of eukaryotic chromosomes [1–4] which play an essential role in the maintenance of chromosome integrity [5–7]. Telomeric DNA in mammals is composed by the tandem repetition of the hexamer (TTAGGG) [2]. In vertebrates, telomeric repeat tracts vary considerably in length between organisms [8]; in humans, double stranded telomeric DNA extends for a few Kb (up to 10) and ends with a G-rich 3’ overhang [9] which folds back and invades the double stranded DNA forming a peculiar structure called T-loop [3]. Telomeric DNA is bound to a specific multiprotein complex called Shelterin, which ensures proper regulation and protection of telomeres [4]. In normal somatic cells telomeres shorten at each cell division due to the inability of DNA polymerase to completely replicate linear DNA [10,11]. Once telomeres reach a critical length, cells enter a non-dividing state [10,11]. Because of its role in controlling cell loss and renewal, progressive erosion of telomeres and cellular replicative senescence are one of the main candidate mechanisms for organismal loss of function with age [7,8]. Rapid deletion in size of telomeric sequences through recombination based mechanisms was also reported in the yeast *Saccharomyces cerevisiae* [12] and in human cells during apoptosis, senescence and tumorigenesis [13]. While the end replication problem provides a general ‘intrinsic’ explanation for telomere shortening, several endogenous as well as extrinsic factors can generate the often observed considerable variation among individuals in telomere length and in the rate at which telomeres shorten. Oxidative and other forms of stress have been shown to accelerate telomere shortening in mammal and bird model species [9–14]. Telomere length and shortening may also depend on genetic differences among individuals, although available estimates of genetic variance in telomere length are few [15].

Because of their fundamental role in cell senescence and susceptibility to broadly diverse factors, telomere dynamics may underpin evolutionary and physiological trade-offs and cause variation in fundamental life history traits such as longevity [16–21]. Several fundamental issues in telomere biology in organisms subjected to natural selection regimes in the wild still remain largely unexplored. First, telomeres have been suggested to undergo faster shortening in early life stages [12,19,22,23]. Some studies have indeed shown that telomere shortening occurs at faster pace among relatively young as compared to older individuals [12,22,24], the most convincing being the evidence from longitudinal analyses, which are not confounded by selection on telomere length. Yet, the temporal scope, relative to the lifespan of individual species, varied considerably among studies. For example, in some bird species which typically complete somatic growth in few weeks/months, telomeres have been shown to shorten most rapidly during the first years of life compared to later life stages [19,21,22,25,26]. Telomere shortening may be expected to be particularly intense during early post-natal stages. For example, severe oxidative stress resulting from accelerated oxidative metabolism may exacerbate telomere shortening during rapid post-natal somatic growth. However, the studies addressing the change in telomere length before growth completion are very few, especially in wild populations [22,23,26,27].

Second, telomeres may be expected to undergo different dynamics depending on individual sex, ultimately resulting in sex variation in telomere length at some life stage (see [28]). Sex-specific telomere length may arise because of fundamental differences in sex determination, with the heterogametic sex being more likely exposed to the expression of unguarded, defective telomere maintenance alleles [29]. In addition, males and females can considerably differ in body size and growth trajectories, and thus in requirements of resources, behaviour, and also in susceptibility to stress factors. The evidence for sex-dependent telomere length and dynamics is equivocal: in birds, some studies have shown sex differences in adult telomere length [26,30–32], whereas others did not show sex-related variation [33,34]. To date, only few studies have identified sex differences in mean length or shortening of telomeres in young individuals before growth completion [14,15,19,23,27].

Third, an association between growth and telomere length may be expected (see [34]), which may be generated by different, not mutually exclusive mechanisms. Hypothetically, if growth and telomere maintenance are both costly activities, a trade-off between growth and telomere length may exist, and individuals with larger somatic growth may undergo larger telomere shortening. However, if variation in individual general physiological state and ecological conditions is wide, no or even positive relationships may be expected, as some individuals will better deal with the telomere maintenance issue without sacrificing growth or even showing relatively high growth rates. Alternatively, growth and telomere dynamics may not be reciprocally linked (see [35]), being both affected by a third factor, such as stress or nutrition. Under this scenario, a positive relationship between telomere length and growth may be expected, because individuals in prime condition may better contrast condition-dependent telomere shortening and also grow at faster pace and to a larger body size. Again, the few existing studies have provided equivocal results by showing variable associations of growth with telomere length [15,23,35,36].

Being sensitive to diverse ecological conditions [23,33,37], telomere length and dynamics may be influenced by social effects, including the number of competitors but also their sex, because males and females may differ in competitive ability and request of food. However, only few studies, with partly conflicting results, have investigated the effect of number of competing siblings or the rank in the brood social hierarchy on telomere length [15,27,35,38], whereas none has addressed the issue of the effect of sex of the competitors on individual telomere length and dynamics.

In the present correlational study of the barn swallow (*Hirundo rustica*), we tackle the issues mentioned above by analyzing telomere dynamics of the offspring over 9 days (from age 7 till age 16 days after hatching), encompassing the period when maximal growth rate is attained [39]. Specifically, we tested if telomere length (TL) changed with age and if age-dependent variation of relative telomere length differed between the sexes, also depending on the number (brood size) and the sex (sibling sex ratio) of the siblings. In addition, we tested if morphological traits reflecting growth covaried with relative telomere length. We expected that telomere length declined with age. Because mean telomere length in fact decreased with age but, contrary to the expectation within-individual variation was found to be positive in a large proportion of individuals, and the frequency distribution of change in TL was bimodal, we scrutinized the data to identify any offspring phenotypic trait that predicted the sign of variation in TL. Due to insufficient theoretical and empirical background, however, we had no unequivocal expectations on sex-dependent telomere shortening or the sex-dependent covariation between morphological traits and telomere length. Finally, we compared telomere length of nestling with that of their attending parents.

## Methods

### Study organism

The barn swallow is a socially monogamous, migratory passerine bird with biparental care of the progeny. Clutches have a modal size of 5 eggs, which hatch after ca. 14 days of incubation. Nestlings fledge 18–21 days after hatching [40]. Nestlings show low to null sexual dimorphism in morphological traits [41]. However, male and female nestlings have been shown to differ in behaviour, with males outcompeting females in scrambling for food, and also being more negatively affected by a stressful competitive nest environment [42–44]. Moreover, nestlings of either sex have negative effects on performance of their siblings when adult, as an increasing proportion of brothers reduces sisters’ fecundity whereas an increasing proportion of sisters reduces phenotypic values at sexually selected traits in adult brothers [45].

### Field procedures

We studied barn swallows breeding in two colonies (farms) located east of Milan (Northern Italy; farm 1: 45° 27' 16.9" N, 9° 19' 30.9" E and farm 2: 45° 27' 38.2" N, 9° 19' 57.0" E) during spring-summer 2013. Nests in rural buildings were regularly inspected starting in early April. The colonies were visited weekly to record breeding events and capture breeding individuals, following well-established procedures (details in [46,47]). All adults were subjected to standard measurements and blood sampling for TL measurement (see below), and sexed according to morphological and behavioural traits. In addition, adults were individually marked with colored rings to identify breeding pairs (details in [46,47]). Breeding males and females were captured on average 15.1 (s.d. = 12.6; n = 11) or 4.8 (s.d. = 19.6; n = 12) days after hatching, respectively. Hatching date was defined as the day when all or most of the eggs in the clutch hatched. Nestling age was expressed as the number of days elapsed from hatching date, while brood size was defined as the number of nestlings present on day 7. Because only one nestling died between age 7 and age 16, brood size at age 7 closely reflects actual brood size during the interval when change in TL was analyzed. At age 7 we individually marked the nestlings with alloy rings and measured body mass (approximation 0.1 g; expressed in g) and tarsus length (approximation of 0.1 mm; expressed in mm). At age 16 we measured again body mass and tarsus length and also wing chord and the length of the growing tail feathers with a ruler (approximation 1 mm; expressed in mm). The data for body mass at age 16 were not used for the purposes of the present study because they mostly reflect the stage in pre-fledging mass recession process of individual nestlings rather than actual body size, which is better indexed by tarsus length. On both day 7 and 16 we collected a blood sample in heparinized capillary tubes after puncturing the brachial vein for molecular sexing using the *CHD* gene according to [48] and relative telomere length measurement (see below). Blood samples were kept in a cool bag while in the field before being taken to the lab where red blood cells were separated by centrifugation and kept frozen at -20°C. The age of 7 days for the first blood sampling was chosen because, according to our decadal experience, it is the earliest age when amounts of blood sufficient for genetic analyses can be obtained with minimal risk of harming the nestlings. Age 16 was chosen for collection of the second sample because we aimed at analyzing morphology at the latest possible stage in the nestling cycle, and age 16 is the latest age when all the nests can be approached without incurring the risk of nestlings prematurely leaving the nest. The interval 7–16 includes the age interval when growth rate of nestling barn swallows is maximal.

### Telomere length measurement

DNA was extracted from 10–20 μl of red blood cells (RBC) using 1 ml TNSE buffer (10 mM Tris HCl, 400 mM NaCl, 100 mM EDTA and 0.6% SDS) and a standard phenol/chloroform method. We measured the quantity and purity of the extracted genomic DNA using a Nanophotometer (IMPLEN). Telomere length was measured by the monochrome multiplex quantitative PCR method (MMQPCR) [49] on a PikoReal 96 thermal cycler (Thermo Scientific). According to this method, telomere length of samples is measured as the ratio (T/S) of the amount of telomeric repeats (T) to the amount of a single copy gene (S), relative to a reference sample. By this method telomere length is evaluated indirectly by measuring the relative number of telomeric repeats in a genome (indicated from now as ‘relative TL’ or T/S ratio). This method can therefore be used to define the trend of telomere length changes without quantifying the size of the loss. Similarly to the TRF (Terminal Restriction Fragment) method, MMQPCR gives T/S ratios of time points that can be used to determine the shortening rates. Since the MMQPCR method evaluates the number of telomeric repeats, it cannot be used when large amounts of telomeric-like repeats at non-terminal sites (Interstial Telomeric Sequences, ITSs) are present in the genome under study [50]. ITSs have been described in all vertebrate species so far analyzed and can be classified, according to sequence organization, into short-ITSs (s-ITSs), composed by short stretches of TTAGGG repeats (up to a few hundreds bp), and heterochromatic-ITSs (het-ITSs), composed by extended blocks of repeats spanning several kilobases and located mainly at pericentromeric regions [51]. While in several species, including humans [52], only s-ITSs are present, in other species, both s-ITSs and het-ITSs have been found [53]. S-ITSs are present at numerous loci (about 100 in the human genome) and, although they are characterized by polymorphism due to variable numbers of tandem repeats [54–56], their effect on telomere length measurements is negligible; on the contrary, het-ITSs might represent a confounding factor. Since the sequence of the barn swallow genome was not available, a preliminary analysis aimed at determining the presence of het-ITSs was carried out by the Terminal Restriction Fragment (TRF) method (S1 Fig) as previously described [57]; since no restriction sites are contained within the TTAGGG repeats, these loci should appear as intense bands [53], while the signal of s-ITS tends to be indistinguishable from the smear due to terminal telomeric repeats [57,58]. No evidence of het-ITSs was observed in the barn swallow DNA and the TRF results were similar to those obtained with human DNA [58]. To better evaluate the presence of het-ITSs in the barn swallow genome, we performed a *Bal*31 assay on the genomic DNA of two unrelated individuals (S2 Fig) following the procedure previously described in [53]. Genomic DNAs from a Chinese hamster cell line (CHO) and from a chicken cell line (DT40), which are known to contain het-ITSs, were used as controls. In CHO and DT40, although we used a concentration of *Bal*31 ten times greater than that used for barn swallow genomic DNA and longer digestion times, we observed intense bands, resistant to *Bal*31 digestion, hybridizing with the telomeric repeat probe. This result confirms that extended blocks of het-ITSs were located at internal chromosome sites. On the contrary, when we digested barn swallow DNA with the *Bal*31 exonuclease we detected a clear reduction in intensity and molecular weight of the smear, indicating that the majority of telomeric repeats detected in these samples were located at chromosome ends. We hypothesize that, if present, ITSs in the barn swallow genome are composed by a small number of repeats (s-ITSs) representing an irrelevant fraction of total telomeric repeats, as in humans [53]. While the MMQPCR method [49] would be unsuitable to measure telomeric repeat content of avian species that are rich in long interstitial telomeric sequence, like chicken [59, 60], it is generally accepted and considered reliable to measure relative telomere length in human cells, despite the presence of s-ITSs. For this reason, we applied the same procedure to the barn swallow.

Finally, another technical issue to be considered is the possible effect of genomic DNA degradation, that may have occurred during blood sample handling and preservation, before DNA extraction. To this regard, it should be pointed out that very little degradation could be observed in our samples following electrophoresis of DNA (S3 Fig). However, to better evaluate the sensitivity of the method to DNA degradation, we carried out MMQPCR reactions with enzymatically digested (DNase I) barn swallow genomic DNA. As shown in S3 Fig, DNA degradation had no significant effect on the measurement of telomeric repeat content.

The sequences of telomeric primers for MMQPCR were previously reported [48] (telg 5’-ACACTAAGGTTTGGGTTTGGGTTTGGGTTTGGGTTAGTGT-3’; telc 5’-TGTTAGGTATCCCTATCCCTATCCCTATCCCTATCCCTAACA-3’). The single copy sequence used as control was a fragment from the 12^th^ exon of the swallow CTCF gene (CCCTC-binding factor zinc finger protein). CTCF was chosen because it is a single copy gene well conserved among vertebrates. Since the swallow genome is not available, a primer pair for the amplification of swallow genomic DNA could be obtained by the alignment of several vertebrate sequences (forward 5’-ATCATTCAGGTTGAAGACCAGA-3’, reverse 5’-GTTATGATTTATTAGCTGTACAGCAGT-3’). The 1.8 kb PCR fragment obtained was then sequenced and swallow specific primers were designed within this sequence. GC clamps were then added to increase the melting temperature of the PCR product. The CTCF primers used were: forward (5’-CCCGCGGCGGGCGGCGCGGGCTGGGCGGCTCCCAATGGAGACCTCAC-3’) and reverse (5’-CGCCGCGGCCCGCCGCGCCCGTCCCGCCCATCACCGGTCCATCATGC-3’); these primers are composed of a swallow genomic sequence and a GC-clamp at the 5’ end (underlined). Since the melting temperature of telomeric and CTCF PCR products are different, both primer pairs could be used in the same reaction.

PCR reactions were prepared using 20 ng of genomic DNA as template, 1x DyNAmo ColorFlash SYBR Green qPCR Master Mix (Thermo Scientific), telomeric primers at a final concentration of 1,000 nM each, CTCF primers at a final concentration of 500 nM each. Three-fold serial dilutions of a swallow reference sample (from 5.5 to 150 ng) were included in each plate to produce a standard curve to measure reaction efficiency and quantify the amount of telomeric repeats and single copy gene in each sample. The average telomere length of the reference sample, as measured by the Terminal Restriction Fragment method [57], was 18 kb (data not shown). All reactions were run in triplicate. Repeatability of relative telomere length estimates, expressed as intra-class correlation coefficient, was 0.77. The coefficient of variation in relative telomere length among plates, as estimated by repeating the reference sample reaction once in each plate was 4.7%.

Cycling parameters for the PCR reactions were: Stage 1: 15 min at 95°C; Stage 2: 2 cycles of 15s at 94°C, 15s at 49°C; and Stage 3: 35 cycles of 15s at 94°C, 10s at 62°C, 15s at 74°C with signal acquisition, 10s at 84°C, 15s at 88°C with signal acquisition.

The PikoReal Software (Thermo Scientific) was used to calculate the amount of telomeric repeats (T) for each sample by interpolation of the quantification Cycle (Cq) into the linear function y = ax + b of the standard curve of the telomeric primers. Similarly, the software calculates the amount of the single copy gene (S) for each sample. Mean values for T and S for each sample were used to calculate the T/S ratios. The software also uses the standard curve to calculate the reaction efficiency as E = [10^(-1/a)^ – 1] × 100, where “a” is the slope of the linear function y = ax + b describing the standard curve [61,62]. The mean reaction efficiencies for both telomere and CTCF amplifications were greater than 90%.

To evaluate the reliability of the MMQPCR, telomere length of 20 samples was also measured by Terminal Restriction Fragment analysis using Sothern blotting, as previously described [57]. Briefly, for each sample, 2 μg of genomic DNA were digested with the restriction enzymes *Rsa*I and *Hinf*I (Thermo Scientific), separated by agarose gel electrophoresis, transferred to nylon membranes and hybridized with a ^32^P-α[dCTP]-labeled telomeric probe. Radioactive signals were detected using a phosphorimager (Cyclone, Packard). The resulting smears were analyzed with the image processing software ImageJ (http://imagej.nih.gov/ij/), and a line graph of optical density (OD) versus DNA migration distance in pixels was generated. By using a calibration curve, based on the migration of a molecular weight (MW) marker, we converted migration distance into MW. Mean TRF length was then calculated, as previously described [63], by applying the formula Mean_TRF_Length = [Σ(OD_i_)/Σ(OD_i_/MW_i_)], where OD_i_ is the optical density at position “i” and MW_i_ is the TRF length at that position. While with this method TRF size distribution from each smear is used to estimate average telomere length, with the MMQPCR method the relative telomeric repeat content, which is expected to be proportional to average telomere length per cell, is calculated [49]. In spite of the differences between the two methods, a good correlation between average telomere length measurement by Southern analysis and the relative telomere length by MMQPCR, was found. The correlation coefficient (r = 0.791, n = 20, P < 0.001) obtained by linear regression analysis was similar to that obtained by previous studies [64,65]. In the text, we will refer to relative telomere length (TL) at the ages of 7 or 16 days as to relative TL_7_ or relative TL_16_, respectively. It should be pointed out that, in higher eukaryotes, where telomere length distribution is broad, due to inter- and intra-cellular variation, mean telomere size comparisons may not provide a complete picture of telomere dynamics and may lead to imprecise evaluation of telomere loss and gain.

### Statistical analyses

We relied on linear mixed models (LMM) while including brood identity as a random effect to account for non-independence of offspring from the same brood [66,67]. Brood identity was retained as a random effect in all analyses. We also initially included a random factor accounting for the effect of colony (= farm). Because exclusion of this term did never significantly change the fit of the model, this random factor was removed from all models. The contribution of random effect factors to the fit of the model was tested using likelihood ratio tests. Degrees of freedom were estimated according to Kenward-Roger’s method. A repeated-measures design was adopted in models of relative TL at different ages by including nestling identity as a within-subject effect. For each nestling we computed a variable ‘sibling sex ratio’ as the proportion of siblings that were male. To avoid the risk of model overparametrization, the analysis of relative TL on morphological trait variation in relation to sex, brood size and sibling sex ratio was split in two models, one where the main and interaction terms of brood size were considered, the other where the terms of sibling sex ratio were included. Because brood size never predicted relative TL or morphological traits, its effect will not be discussed further and we will focus on the models including sex and sibling sex ratio.

We objectively assessed deviation of change in relative TL from unimodalilty using Hartingan’s Dip test, as implemented using *diptest* package in R 3.0.1 (R Core Team 2013), and applying 5000 replicates in Monte Carlo simulation. Because a non-negligible proportion of nestlings showed an increase, rather than the expected decrease in relative TL, we also set a cutoff at change in relative TL = 0 and checked if the frequency distribution conformed to a bimodal distribution using Ashman’s D statistic, whereby D > 2.0 is considered to reflect bimodality. To compare binomial linear mixed models of the sign of change in relative TL (coded as 0: negative change; 1: positive change) including or, respectively, excluding the random effect of brood, we used likelihood ratio test, where marginal likelihoods were estimated using Laplace approximation [68].

To resolve among-broods from within-broods effects, in the analyses of nestling morphological traits we adopted the approach suggested by [69], which consists in centering the independent variable(s) around the brood (“subject” in [69] terminology) mean. The rationale of this procedure, and the importance of avoiding generalizing within-subject (i.e. within-brood) to among-subjects (i.e. among-broods) effects, or vice versa, in linear mixed models is fully described in [69]. Thus, for each nestling we calculated the value of two new variables: MEAN-TL which equaled the brood relative TL mean (and was thus invariant within brood), and DEV-TL, defined as the nestling relative TL value minus the brood mean.

In linear mixed model analyses we first included the two way interaction effects among factors and covariates. The non-significant interactions were then removed.

Relative TL estimate was not available for one sexed nestling at age 7, while tarsus length could not be measured in one brood (4 nestlings) at age 16. Two nestlings could not be sexed. In all analyses, the largest available sample was used.

Because relative TL measured on blood samples collected from social mothers and fathers (see Field procedures) did not significantly vary according to date of blood sampling of females (r = -0.15; date of blood sampling of males was invariant), or with breeding stage at which blood was sampled (i.e. the difference in days between blood sampling and egg hatching date) (males: r = -0.22; females: r = -0.27; all P values > 0.39), the effects of these variables on parental relative TL was not considered further.

In the comparisons between relative TL of the nestlings and of their parents we considered only the parents that were 1-year old, in order to avoid any confounding effect of age on relative TL.

Throughout the text, “F” indicates the value of Fisher’s F statistic, P is the probability value associated to the relevant statistic, and parameter estimates are given with their associated standard error. All relevant data used in the statistical analyses have been reported in S1 Dataset. Statistical analyses were run using SPSS 13.0 or SAS 9.3 statistical packages.

### Ethics statement

We studied barn swallows breeding in two farms located east of Milan (Northern Italy; farm 1: 45° 27' 16.9" N, 9° 19' 30.9 "E and farm 2: 45° 27' 38.2" N, 9° 19' 57.0" E). When removed from their nest, nestlings were kept in a safe and warm place. At each measurement session each nestling was handled only for few minutes and nests were never left without at least one nestling inside to avoid parental desertion. Adult birds were captured with mist-nets, extracted from the nets within 10 min of capture, kept safely in cloth bags, blood-sampled and released as soon as possible (usually within 1 h), following standard capture and handling techniques aimed at minimizing adverse effects. Blood samples (50–100 μl) were collected by slightly puncturing the brachial vein with sterile needles and the puncturing site was carefully disinfected. No obvious negative consequences of handling nestlings or capturing adults were detected. Capture, handling and blood sampling of barn swallows was authorized by Regione Lombardia (Decreto n° 2141, issued on March 9, 2011). As no manipulative experiments were carried out, no approval from an ethical committee was required for this study.

## Results

Relative TL was estimated on a total of 60 sexed nestlings from 15 broods (13 and 2 from either of two farms). The frequency of males (n = 31) exceeded that of females (n = 29), yielding a slight, non-significant male-biased sex ratio (proportion of males: 0.517; asymptotic P from binomial test: 0.90). Brood sex ratio ranged between 0 and 1 (mean: 0.53) (S4 Fig). Its variance (s.d. = 0.25) was closely similar to that recorded in a large sample of 621 broods from 11 years in the same population (s.d. = 0.28; our unpublished data). Hence, the broods included in the sample were representative of the variation in brood sex ratios in the population we studied. In the sample of 60 sexed nestlings, there was ample variation in the sex ratio of the siblings (S5 Fig).

**Fig 1:**
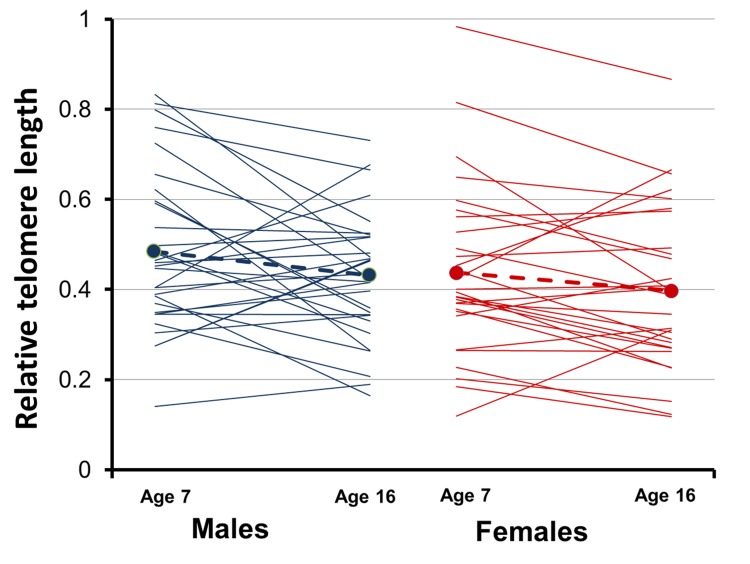
Relative telomere length (T/S ratio: mean ± s.e.) of male and female barn swallow nestlings 7 or 16 days after hatching. Values for relative telomere length at day 7 and day 16 for each individual are shown as a line.

We first analyzed variation of relative TL between age 7 and 16 in a LMM where we included sex, age and sibling sex ratio as predictors together with their two-way interactions. A simplified model excluding the non-significant two-way interactions showed that relative TL decreased significantly, by ca. 10%, between age 7 and age 16 (Fig 1). Hence, there was a decrease in relative TL during the 9 days including the phase of maximal growth of nestling barn swallows, and such decline did not depend on sex, brood size or social nest environment in terms of sex ratio (see Statistical analyses).

**Fig 2:**
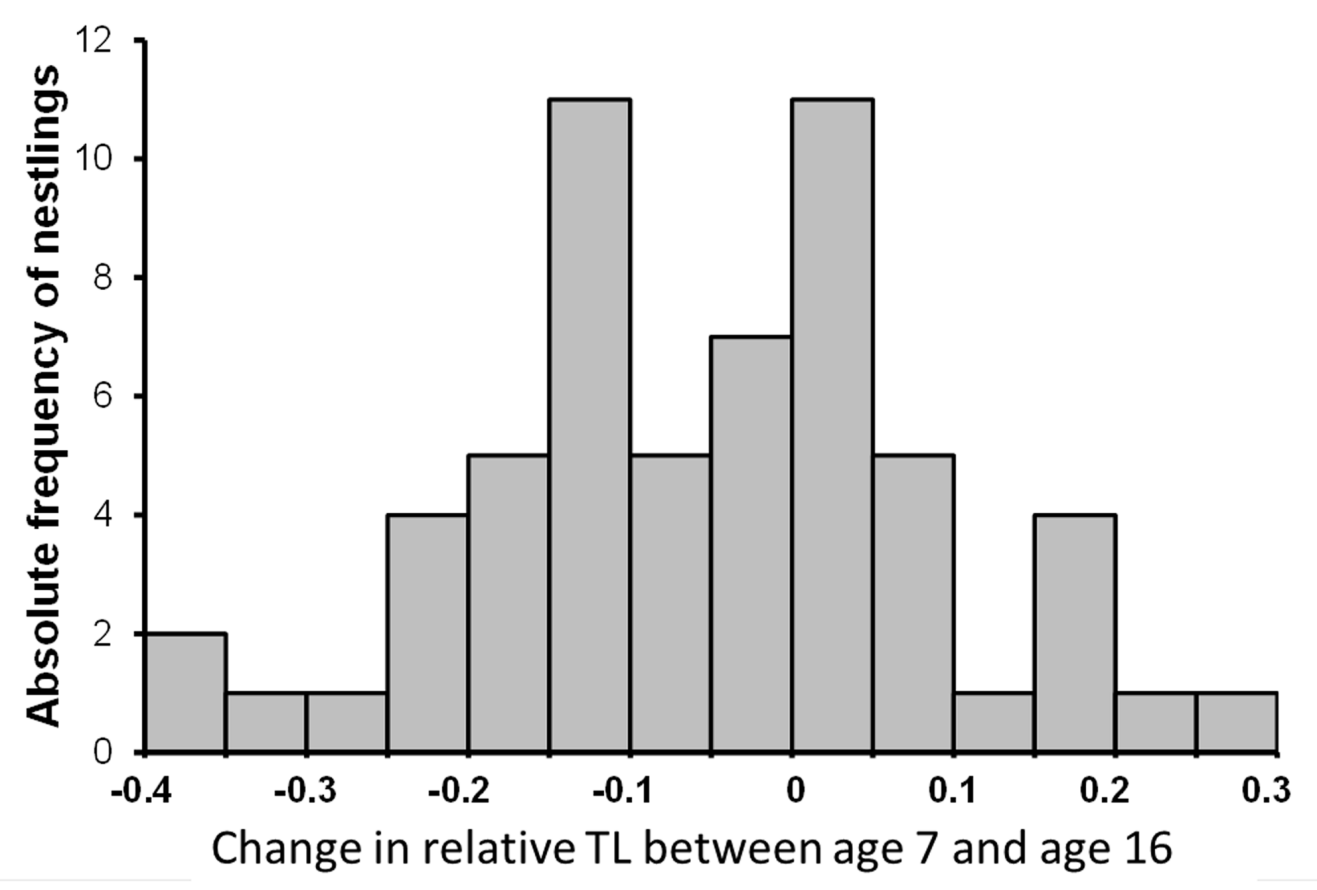
Frequency distribution of change in relative TL between the age 7 and the age 16. The distribution is bimodal.

Relative TL on average declined between age 7 and age 16 after hatching, but 39% of the nestlings showed a positive, rather than negative change in relative TL. Hartingan’s Dip test for unimodality indicated that there was no significant deviation of the frequency distribution of change in relative TL between age 7 and age 16 days after hatching from an unimodal distribution (D = 0.048, P = 0.385). However, visual inspection of the frequency distribution (Fig 2) and the result of Ashman’s D test for bimodality while applying change in relative TL = 0 as a cutoff between two mixed unimodal distributions was consistent with the hypothesis that the frequency distribution of the changes in relative TL consisted of two separate subpopulations of nestlings that underwent either an increase or a decreased in relative TL (Ashman’s D = 2.59). We thus scrutinized the data in order to identify the source of variation in the sign of change in relative TL with age. First, we tested if such heterogeneity occurred among broods in a likelihood ratio test comparing the goodness of fit of a binomial LMM including brood as a random factor to the fit of a model including only the random intercept effect. Because the likelihood ratio test showed a non-significant effect of brood (χ^2^_1_ = 0.00; P > 0.99). We then tested if the sign of change in relative TL of individual nestlings, coded as a binary response variable (0: decrease in relative TL; 1: increase in relative TL), was predicted by the nestling phenotypic traits that we measured at the age 7. In separate binomial LMM with brood as a random effect, the sign of the change in relative TL was not predicted by the effect of brood size (F_1,44_ = 0.66, P = 0.423), sex (F_1,43_ = 0.48, P = 0.490), sibling sex ratio (F_1,43_ = 0.00, P = 0.981), tarsus length (F_1,43_ = 0.46, P = 0.503), body mass (F_1,43_ = 1.27, P = 0.267) or hatching date (F_1,44_ = 0.45, P = 0.505). Thus, none of the phenotypic traits that we measured on the offspring at the age of 7 predicted whether relative TL subsequently increased or, conversely, decreased.

Log-likelihood ratio tests showed that, compared to the null (intercept-only) model, the LMM including the random effect of nestling identity provided a better fit (*χ* ^2^_1_ = 33.3, P < 0.001). The fit of the model increased further when brood identity was also included as a random effect (*χ* ^2^_1_ = 5.8, P = 0.016). Thus, relative TL varied both among broods and among nestlings.

**Fig 3:**
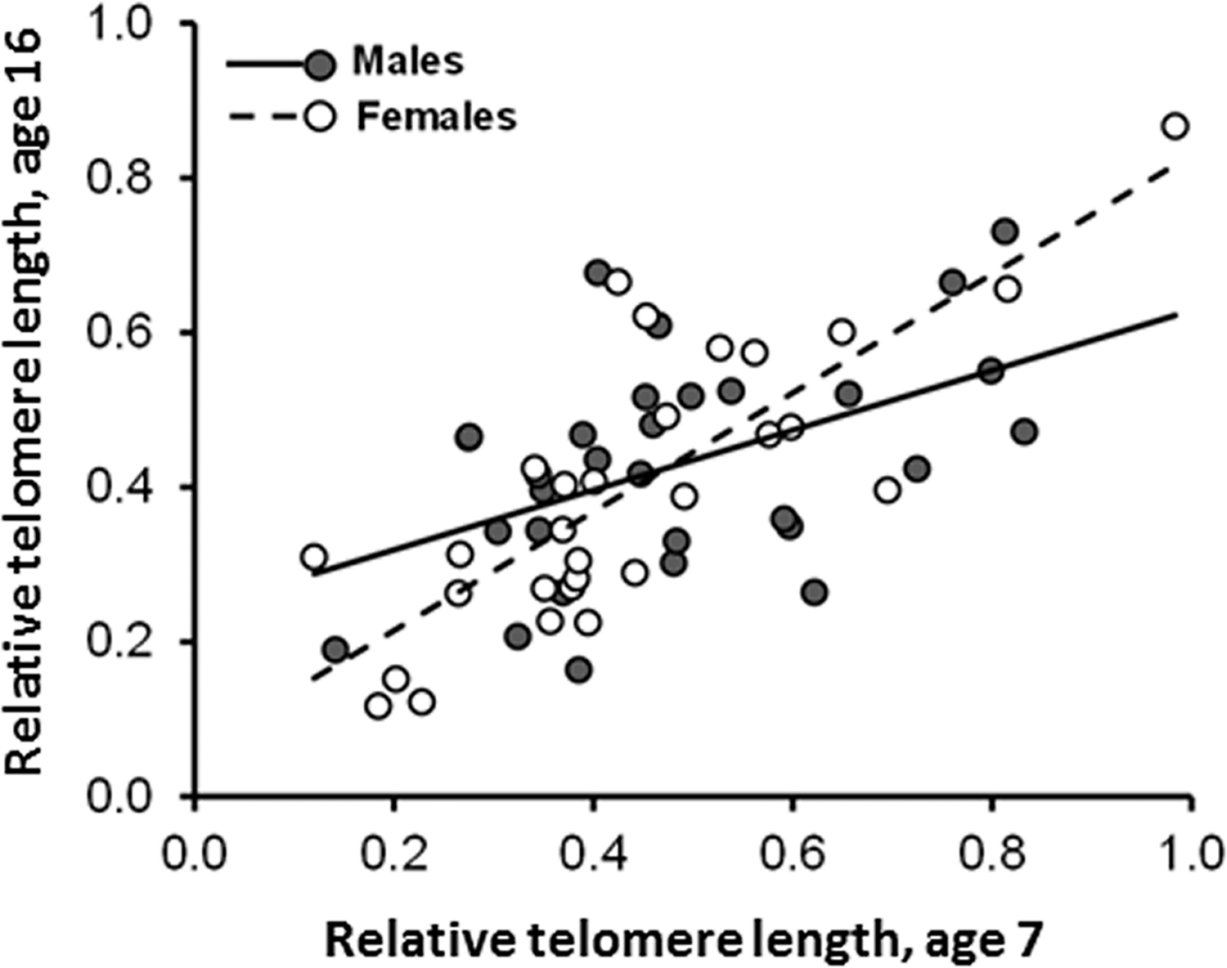
Relative telomere length (T/S ratio) of male and female barn swallow nestlings 16 days after hatching in relation to relative telomere length 7 days after hatching. The slope of the relationship for females (n = 29) was significantly larger than for males (n = 30).

In a LMM with brood as a random effect, relative TL_16_ positively but differentially covaried with relative TL_7_ in either sex (interaction between sex and relative TL_7_: F_1,55_ = 4.92, P = 0.031; the variances of relative TL were homogeneous between males and females at both sampling ages: Levene test: P > 0.17). This was the case because the slope of the relationship between relative TL_16_ and relative TL_7_ was approximately twice as large in females (0.768 (0.119); t_55_ = 6.47, P < 0.001) as compared to males (0.387 (0.124); t_55_ = 3.11, P = 0.003) (Fig 3). This result may appear to contradict the finding of no sex by age interaction effect on relative TL in a repeated-measures LMM (see above and Table 1). Rather, and interestingly, the two analyses combined imply that on average female and male nestlings experienced similar average telomere shortening from age 7 till age 16, but among individuals that at the age of 7 days had large relative TL, at the age of 16 females had larger relative TL as compared to males. Conversely, among individuals with small relative TL at the age of 7, at the age of 16 males had larger relative TL than females. Indeed, in a LMM the change in relative TL, computed as the difference between relative TL_16_ minus relative TL_7_, was differentially associated with relative TL at age 7 in males and females (interaction between sex and relative TL_7_: F_1,55_ = 4.92, P = 0.031).

**Table 1:** Repeated-measures LMM analysis of relative telomere length at day 7 or 16 after hatching in relation to sex and brood size.

	F	df	P	Estimated marginal means (s.e.)
Sex	1.00	1, 57.2	0.322	Males: 0.458 (0.028); Females: 0.418 (0.029)
Age	6.64	1, 58.5	0.013	Day 7: 0.462 (0.022); Day 16: 0.414 (0.022)
Sibling sex ratio	0.50	1, 56.9	0.484	-

Brood and nestling identity were included as a random effects. Two separate models both including sex and age, and also brood size or sibling sex ratio, respectively (see Statistical analyses) did not disclose significant interaction effects. The effect of age was significant also after excluding the effect of sibling sex ratio. The analysis is based on 119 relative TL estimates from 60 nestlings, because the datum for one nestling at age 7 was missing.

Paired t-tests indicated that within-brood mean relative TL of the nestlings at age 16 did not differ from that of their parents (relative TL of parental father minus mean offspring TL: 0.031 (0.116), paired t test: t_7_ = 0.264, P = 0.800; TL of parental mother minus mean offspring TL: 0.017 (0.084), t_9_ = 0.208, P = 0.840). Thus, parents did not have shorter telomeres compared to their offspring.

Nestling morphology was analyzed in relation to relative TL recorded at the same age when the traits were measured in LMM with sex, MEAN-TL, DEV-TL, sibling sex ratio and their two way interactions as predictors. The models of body mass at age 7 and of tarsus length at both age 7 and 16 did not disclose any significant interaction or main effects (Table 2).

**Table 2:** Linear mixed models of nestling morphology in relation to relative telomere length.

	F	df	P	Coefficients (s.e.)
*Body mass*, *age 7*				
Sex	1.22	1, 27.7	0.279	
MEAN-TL_7_	0.57	1, 12	0.465	
DEV-TL_7_	0.62	1, 42.2	0.436	
Sibling sex ratio	0.02	1, 16.5	0.902	
*Tarsus length*, *age 7*				
Sex	1.34	1, 30.3	0.256	
MEAN-TL_7_	0.02	1, 12	0.901	
DEV-TL_7_	0.89	1, 42.4	0.350	
Sibling sex ratio	0.21	1, 15.9	0.656	
*Tarsus length*, *age 16*				
Sex	0.98	1, 30.5	0.331	
MEAN-TL_16_	0.78	1, 11.5	0.395	
DEV-TL_16_	0.30	1, 39.9	0.584	
Sibling sex ratio	0.02	1, 15.2	0.898	
*Wing length*, *age 16*				
Intercept				42.81 (4.44)
Sex	12.92	1, 50.3	0.001	Males: -17.61 (4.90), Females: 0*
MEAN-TL_16_	19.40	1, 11.9	<0.001	0*
DEV-TL_16_	16.02	1, 47.9	<0.001	0*
Sibling sex ratio	2.82	1, 15.7	0.113	-4.30 (2.56)
Sex × MEAN-TL_16_	10.32	1, 49.3	**0.002**	Males: 56.16 (10.99)^a^, Females: 19.32 (9.98)^b^
Sex × DEV-TL_16_	10.30	1, 51.5	**0.002**	Males: 45.25 (10.52)^c^, Females: 13.81 (6.79)^d^
DEV-TL_16_ × Sibling sex ratio	13.89	1, 49.7	**<0.001**	-51.06 (13.70)
*Tail length*, *age 16*				
Intercept				21.95 (3.92)
Sex	5.67	1, 50.6	0.020	Males: -10.42 (4.37), Females: 0*
MEAN-TL_16_	18.40	1, 12.3	0.001	0*
DEV-TL_16_	3.02	1, 48.2	0.089	0*
Sibling sex ratio	1.63	1, 16.3	0.220	-2.86 (2.24)
Sex × MEAN-TL_16_	4.12	1, 49.7	**0.048**	Males: 42.55 (9.70)^e^, Females: 21.77 (8.41)^f^
Sex × DEV-TL_16_	11.32	1, 51.7	**0.002**	Males: 26.14 (9.38)^g^, Females: -3.24 (6.09)^h^
DEV-TL_16_ × Sibling sex ratio	4.69	1, 50	**0.035**	-26.48 (12.23)

For wing and tail length, the parameters are explicitly given to allow calculation of fitted phenotypic values. Non-significant interaction terms were removed from the final models (see Statistical analyses). Coefficients for non-significant terms are not reported. Bolded P-values are discussed in the text.

*: these parameters are set to 0 as they are redundant.

a: t_25.3_ = 5.11, P < 0.001

b: t_16.5_ = 2.02, P = 0.060

c: t_51.4_ = 4.30, P < 0.001

d: t_40.7_ = 2.03, P = 0.049

e: t_51.6_ = 4.39, P < 0.001

f: t_17.2_ = 2.59, P = 0.019

g: t_51.6_ = 2.86, P = 0.007

h: t_41.2_ = -0.53, P = 0.597

**Fig 4:**
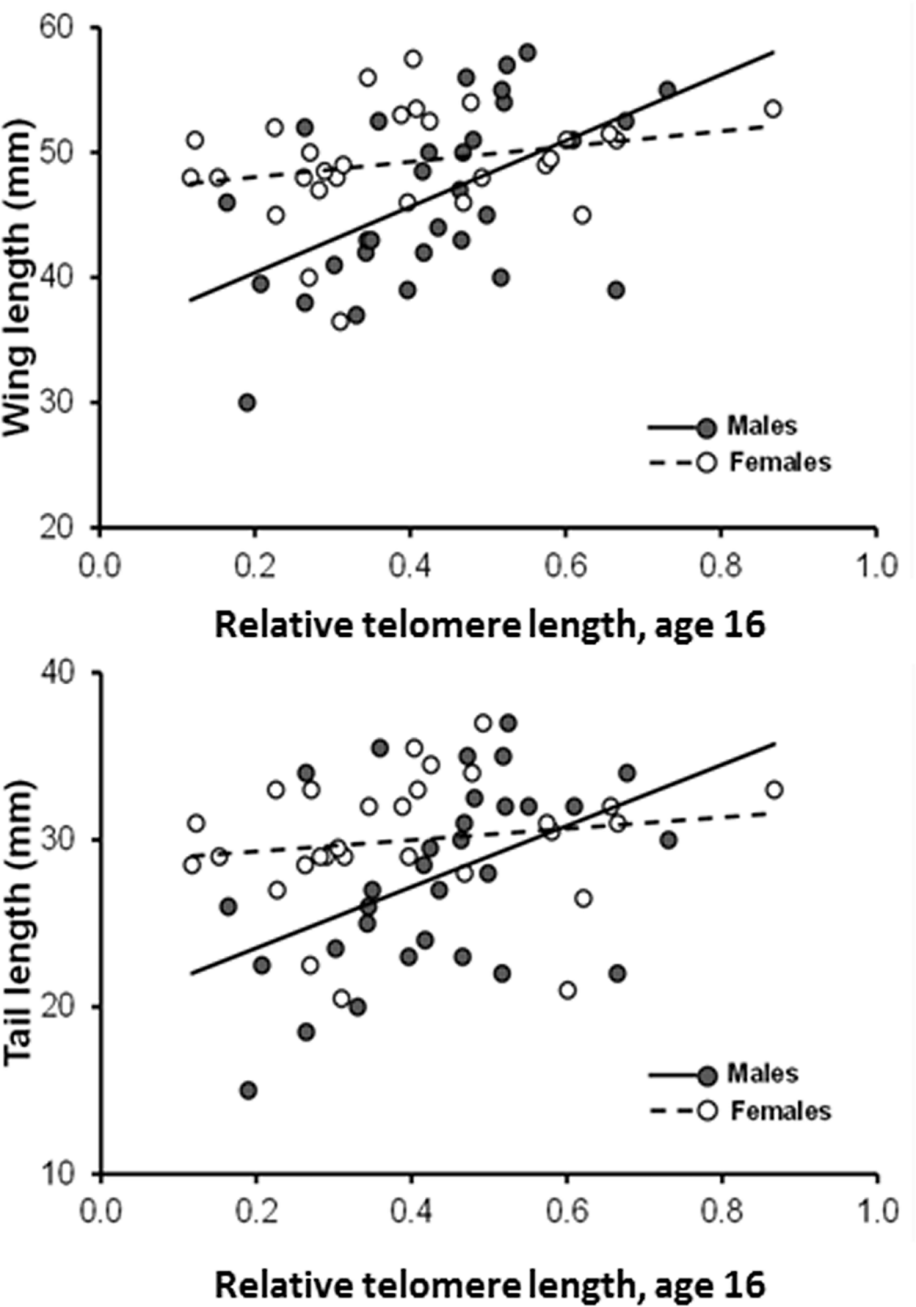
Length of the wing chord (upper panel) or tail length (lower panel) at age 16 days in relation to relative telomere length (T/S ratio) on the same day. For wing length, the relationship was significantly positive for both males (n = 31) and females (n = 29), but was steeper in males. For tail length, the relationship for males (n = 31) was significantly positive and significantly steeper than in females (n = 29).

Both wing and tail length differentially covaried with DEV-TL_16_ (Table 2; bolded P-values). Wing length significantly increased with relative TL_16_ among both males and females, though more steeply so in the former (Table 2; Fig 4). Tail length increased with relative TL_16_ in males, whereas it was not significantly associated with relative TL_16_ in females (Table 2; Fig 4). Both wing and tail length increased with MEAN-TL_16_, while controlling for DEV-TL_16_ in both males and females, and more steeply so in males. This implies that, independent of relative TL_16_ of individual nestlings relative to their siblings, nestlings in broods with larger mean relative TL also had larger plumage phenotypic values (Table 2).

In addition, both plumage traits were predicted by the DEV-TL_16_ by sibling sex ratio interaction (Table 2). The coefficients of the models in Table 2 imply that both traits increased with increasing DEV-TL_16_ but the increase was steeper when the proportion of brothers decreased. The results of the analyses where morphological traits at age 16 were modelled in relation to relative TL_7_ were qualitatively similar to those obtained based on relative TL_16_ (details not shown).

## Discussion

In this correlational study we analyzed sex-dependent variation of relative TL and telomere shortening of nestling barn swallows and whether any covariation between morphological traits and relative TL or telomere shortening depended on social effects. Telomere length measurements were carried out in peripheral blood; these are considered to provide a good evaluation of telomere length and dynamics in the entire organism [70,71]. Particularly relevant in this context are the results obtained by Reichert and collaborators [72] showing, in adult zebra finches, a relationship between telomere length in red blood cells and in different somatic tissues. Relative TL decreased within individual nestlings over the mid nestling period. Individual nestlings were consistent in their relative TL during the nestling period, but the positive relationship between relative TL at the ages of 7 and 16 days differed in slope between males and females. Values of change in relative TL during the nestling stage were bimodally distributed, implying a different pattern of relative TL variation. In addition, plumage growth, as reflected by both tail and wing feather length, increased with relative TL but more so among males than females. Finally, the association between the increase in nestling body size, as reflected by tarsus length, or plumage traits and relative TL depended on the proportion of nest mates that were male.

Telomere shortening may be especially rapid early in life. However, the temporal scale at which age-dependent relative TL dynamics have been resolved in longitudinal analyses has varied considerably among studies, as only few of them have looked at change in relative TL before growth completion/independence [19,22,26,28]. Here we demonstrate considerable (ca. 10%) shortening of telomeres over the mid part of the nestling period of barn swallows, lasting only 9 days. Mean relative TL of 16 days old nestlings did not differ from that of their 1-year-old parents, suggesting that telomere shortening during the nestling period accounts for a large part of total lifetime telomere shortening [12,19,22]. The alternative possibility that shortening of telomeres is intense also after fledging before the first breeding season, and that strong selection on relative TL occurs after growth completion before recruitment into the breeding population cannot be ruled out. Previous studies have shown that telomere length and dynamics are, at least to some extent, chromosome-specific [73–77], thus, the intense shortening observed for some individuals may reflect changes at subsets of telomeres.

Unexpectedly, the values of change in relative TL increased, rather than decreased, in approximately 40% of the individuals. Considerable variation in the extent of change in relative TL with age is also apparent from some previous studies where individual-level longitudinal data are presented [37], and some studies also present evidence that a variable proportion of individuals appear to undergo an increase, rather than a decrease in TL with age (see [18,23,78]). The frequency of apparent increase in TL in previous studies is difficult to estimate because of the potentially confounding effect of selection. This is the case because mortality during the study (i.e. between the time of the first and of the subsequent measurements of TL) may not be random with respect to TL, as individuals with initially shorter telomere may be expected to have smaller life expectancy. Yet, the relative frequency of individuals showing an increase in TL seem to range between values close to 0 [18] to large [78]. In the present study, mortality between the age of 7 and the age of 16 after hatching was about 2%. Thus, the estimated frequency (= 39%) of individuals that apparently increased their relative TL is not unbiased by mortality dependent on TL. When we scrutinized our data in an attempt to identify which offspring traits predicted increase rather than decrease in relative TL, however, we found no effect of individual-level traits (nestling morphology and sibling sex ratio) or brood-level (brood identity and brood size). Thus, factors predicting decrease or, respectively, increase in relative TL remain unidentified in the present study. Because no brood-level variation in the relative frequency of nestlings that showed opposite signs of variation in relative TL were found, we may speculate that such differential variation arose either because of individual genetic differences in within-family telomere dynamics and/or because of variation in early maternal effects as those mediated, for example, by egg biochemical quality. Egg corticosterone, for example, can cause reduction in telomere length [14]. Variation in corticosterone concentrations among sibling eggs, which has been shown to occur in barn swallows according to laying order, could have caused persistent differential effects in telomere shortening during the nestling period. The mechanism of telomere elongation in some individuals remains elusive since to our knowledge, no data concerning telomere elongation or telomerase activity in blood cells from barn swallow are available. A previous work by Haussmann and collaborators [79] detected telomerase activity in several tissues from tree swallow, including bone marrow, and associated the level of activity with the rate of telomere shortening in erythrocytes. Interestingly, in blood cells of some long-lived bird species, an increase in telomere length with age rather than a decrease was observed in some individuals [26,32,33,80]. Recently, Lin and collaborators described a slight telomere length increase in peripheral mononucleate blood cells in a human subpopulation [81]. In addition, it was reported that, in human lymphocytes, telomerase is activated upon antigen receptor activation-induced cellular proliferation [82–84], which generally occurs during inflammation or infection. The biological significance and the molecular mechanism of elongation, has to be established. These observations suggest that more attention should be paid in future studies to the identification of ‘clusters’ of individuals that show different patterns of temporal variation in telomere length and of the factors that lead to such differential patterns of change in TL.

We did not identify any difference in mean relative TL nor in change in relative TL during the nestling period between male offspring and their female siblings. These results are consistent with those of most of previous studies investigating the sex-related difference in relative TL or in change in mean relative TL during the nestling period [14,15,23,27,32]. Overall, nestlings having longer telomeres at day 7 also had longer telomeres at age 16 according to [22,23]. Within-sex variation in relative TL between day 7 and day 16 was marked. Yet, and intriguingly, we found a statistically significant difference in the change in relative TL from day 7 to day 16 according to relative TL at age 7 between male and female nestlings. Specifically, the slope of the relationship between relative TL_16_ and relative TL_7_ was steeper in females than in males, implying that among the individuals with small relative TL_7_, females had smaller relative TL_16_ than males, whereas among individuals with large relative TL_7_, females had larger relative TL_16_ relative to males. Conversely, among individuals with small relative TL_7_, females ended up with smaller relative TL_16_ than males. This suggests complex sex-dependent telomere dynamics whereby shortening depends on initial TL but differentially so in either sex. This result indicates that no sex-related variation in relative TL can in fact be underpinned by differences in telomere dynamics between the sexes. Females with initially short telomeres may be more sensitive to their nest environment, and undergo larger telomere shortening than males. Initially (i.e. at age 7) small relative TL may itself reflect more intense telomere shortening at previous pre- or early post-hatching stages, possibly as consequence of a stressful environment within the brood. If this is the case, the present results suggest that females starting their growth in an adverse environment may suffer from oxidative stress, as suggested by a previous study of zebra finch (*Taeniopygia guttata*) nestlings after experimental elevation of the stress hormone corticosterone during rearing [14]. Indeed, several studies have provided convincing evidence that poor early growth conditions can result in high levels of oxidative stress [36,85,86], leading to an increased telomere erosion [9,10,36,87]. On the other hand, variation in telomere length in early life stages may be unrelated to post-natal environmental conditions and diverse forms of stress, and rather reflect heritable variation in telomere length and/or telomere dynamics.

We hypothesized that telomere dynamics could depend on the number and sex of the nest mates, as these can influence the level of social stress and nutritional conditions. However, there was no hint of any effect of brood size. This evidence is consistent with a previous experiment where no variation in nestling TL was observed among collared flycatcher (*Ficedula albicollis*) broods whose size had been increased, reduced or not manipulated [15].

Body size, as reflected by tarsus length, and body mass did not covary with relative TL in both sexes. However, both plumage traits we measured were positively predicted by relative TL_16_. By applying within-subject centering to mixed models of nestling phenotypic traits, we showed that these relationships held both at the within- and at the among-broods levels. Thus, not only nestlings with larger relative TL_16_ relative to their brood mates had larger tail and wing feather length at a given age, but, independently of any within-brood variation in relative TL_16_, broods with larger average relative TL_16_ did also host nestlings with larger plumage traits. Both among- and within-brood effects can be interpreted in two alternative perspectives, which rest on different assumptions on the causal links between TL and growth. First, some individuals can afford larger growth because of their larger TL. Alternatively, the same conditions that promote growth also cause retention of larger TL. For example, better nutritional conditions may both reduce telomere shortening and allow for faster plumage growth and thus feather length at a given age, producing a positive association between phenotypic values and TL both at the within and at the among-broods level.

The relationships between plumage traits and relative TL_16_, which were positive in both sexes, were significantly steeper in males than in females, implying that male nestlings had larger increase in phenotypic values per unit increase in relative TL_16_. These findings are partly consistent with a previous study of adult barn swallows, showing a positive correlation between TL at nestling stage and body size at adulthood in males, even if no relationship with feather length was noticed [34]. This discrepancy may be due to feather length in breeding adults being strongly determined by environmental conditions at the wintering grounds, where molt of wing feathers occurs [88].

While relative TL_16_ was not predicted by the sex ratio of the nest mates, the association between plumage traits and individual relative TL_16_ varied according to sex ratio. Plumage traits were larger when DEV-TL_16_ increased, but more so with increasing proportion of female siblings. The fact that social effects, in terms of sex ratio of the siblings, interfered with the expression of the association between phenotype and TL argues in favor of the hypothesis that the positive association between feather traits and relative TL_16_ reflects an association of both variables with general condition as influenced for example by individual nutritional state, which can be depressed by growing in a male-biased brood [48].

Thus, our study shows that subtle difference exist in telomere dynamics between the sexes which lead to differential telomere shortening according to initial relative TL in either sex but not to a difference in mean relative TL or relative TL change during the nestling period between the sexes. In addition, growth of plumage traits is differentially predicted by relative TL in either sex, possibly because of a differential effect of rearing environment on growth and telomere dynamics. Finally, our study suggests that the relationship between individual phenotype and telomere dynamics may be also affected by sex-dependent sib-sib competition.

## Supporting Information

S1 Dataset The file summarizes all the relevant data that have been used in the statistical analyses. (XLS)

S1 Fig Terminal Restriction Fragment (TRF) analysis by Southern blotting in 10 barn swallows. *Hind*III-digested Lambda DNA was used as molecular weight marker, the size (kb) and the positions of the markers are indicated on the left. No intense bands corresponding to het-ITSs were detected.
(TIF)

S2 Fig Hybridization of a telomeric-repeat probe to Chinese hamster (CHO), chicken (DT40) and barn swallow genomic DNA digested with *Bal*31 exonuclease. High molecular weight genomic DNA was prepared by standard phenol/chloroform method from a Chinese hamster cell line (CHO), a chicken cell line (DT40) and from red blood cells collected from two barn swallows. Genomic DNAs were digested with either 0.05 (CHO and DT40) or 0.005 (barn swallow) units of *Bal*31 (Takara) per μg of DNA in 1x Bal31 Nuclease Buffer (Takara) at 30°C. Aliquots containing 3 μg of digested DNA were withdrawn from all reactions after 0, 5, 10 and 30 minutes. Additional aliquots of digested CHO and DT40 genomic DNAs were withdrawn after 60 and 120 minutes. Reactions were blocked by the addition of EGTA (final concentration 20 mM) and incubation at 65°C for 10 minutes. After phenol-chloroform extraction, DNAs were ethanol-precipitated, resuspended in water and digested for 12 hours with 10 units of *Hinf*I (Thermo Scientific) per μg of DNA. Digested DNA was electrophoresed in 1% agarose gel, denatured and transferred to a nylon membrane (Amersham Hybond-N, GE Healthcare). Membranes were then hybridized with a ^32^P-α[dCTP]-labeled telomeric probe and exposed to autoradiografic films as previously described [56]. *Hind*III-digested lambda genomic DNA and GeneRuler 1 kb DNA Ladder (Thermo Scientific) were used as molecular weight markers, the size (Kb) and the positions of the markers are indicated on the left.
(TIF)

S3 Fig Relative telomeric repeat content of DNase I-digested swallow genomic DNA measured by MMQPCR. (A) Agarose gel electrophoresis of barn swallow genomic DNA digested with DNase I. (B) Relative telomeric repeat content. Barn swallow genomic DNA was digested with 0.001 units of RNase-free DNase I (Thermo Scientific) per μg of DNA in 1x Reaction Buffer (Thermo Scientific) at 37°C. Aliquots containing 2 μg of DNA were withdrawn after 0, 0.5, 2 and 5 minutes. Digestion was blocked by the addition EDTA to a final concentration of 5 mM and incubation at 65°C for 10 minutes. Degradation was checked by electrophoresis in 1% agarose gel. Digested DNA was ethanol-precipitated and resuspended in 1x nTE. Relative telomeric repeat content was measured by MMQPCR as described in Methods. The relative telomeric repeat content of the untreated sample (0 minutes) was used as reference.
(TIF)

S4 Fig Frequency distribution of the sex ratios of the broods included in the sample. (TIF)

S5 Fig Frequency distribution of the sex ratios of the siblings for any particular nestling. For example, a sex ratio of siblings = 0.5 indicates that half of the siblings of a particular nestling were male and half were female.
(TIF)
