# Supplementary figures and images for: Early-Life Telomere Dynamics Differ between the Sexes and Predict Growth in the Barn Swallow (Hirundo rustica)
Source: PLoS One. 2015 Nov 13;10(11):e0142530. doi: 10.1371/journal.pone.0142530 (PMC4643985; doi:10.1371/journal.pone.0142530)

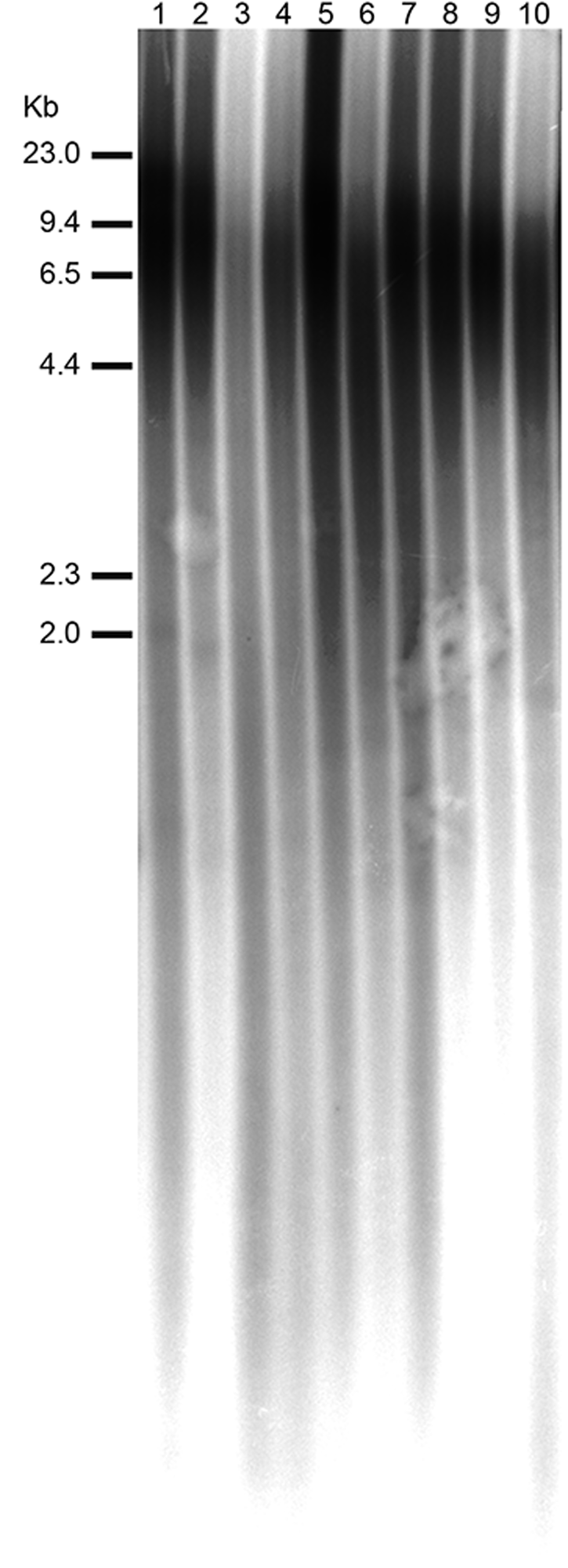

Supplement: S1 Fig — HindIII-digested Lambda DNA was used as molecular weight marker, the size (kb) and the positions of the markers are indicated on the left. No intense bands corresponding to het-ITSs were detected. (TIF) [file pone.0142530.s002.tif]

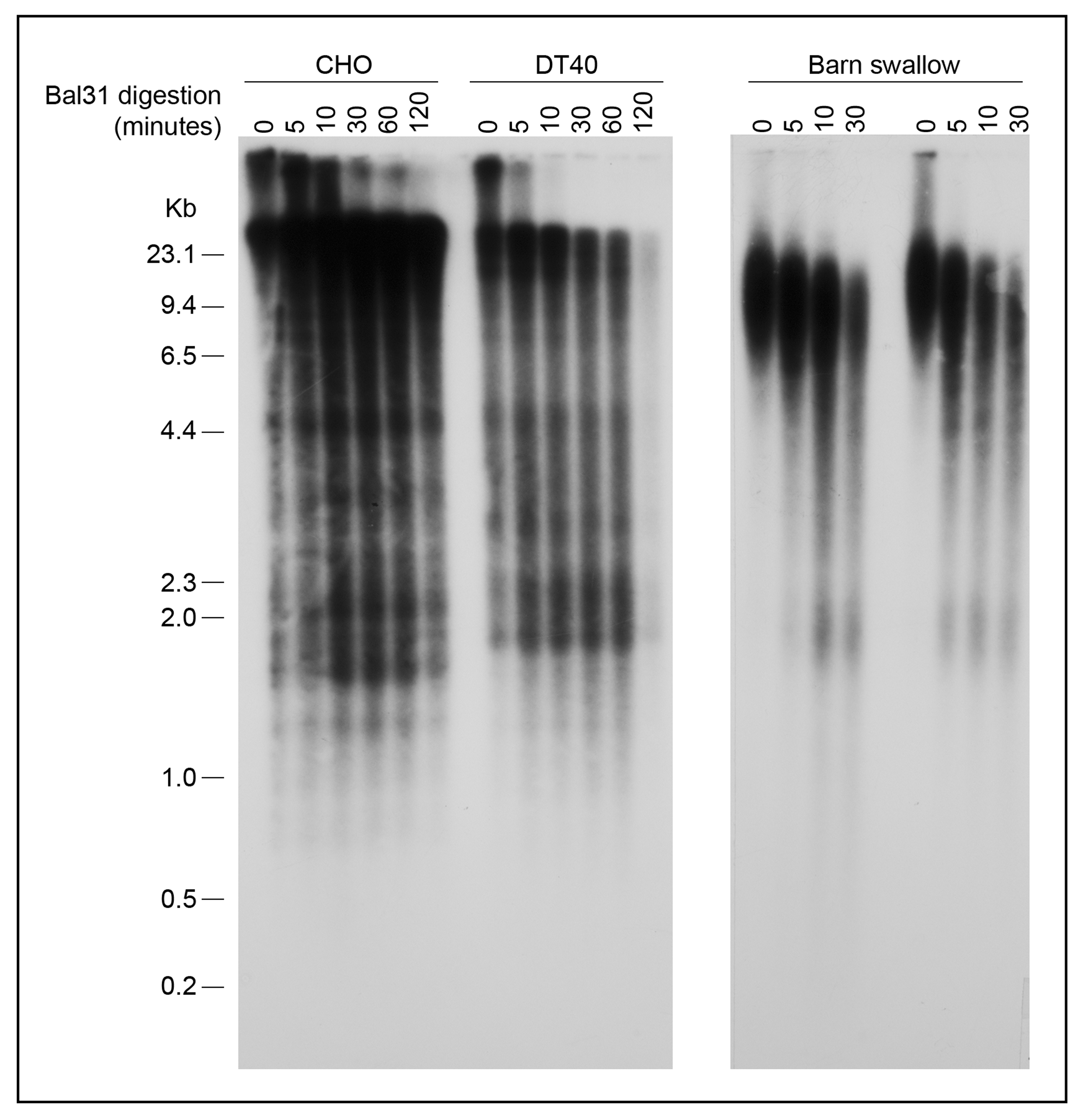

Supplement: S2 Fig — High molecular weight genomic DNA was prepared by standard phenol/chloroform method from a Chinese hamster cell line (CHO), a chicken cell line (DT40) and from red blood cells collected from two barn swallows. Genomic DNAs were digested with either 0.05 (CHO and DT40) or 0.005 (barn swallow) units of Bal31 (Takara) per μg of DNA in 1x Bal31 Nuclease Buffer (Takara) at 30°C. Aliquots containing 3 μg of digested DNA were withdrawn from all reactions after 0, 5, 10 and 30 minutes. Additional aliquots of digested CHO and DT40 genomic DNAs were withdrawn after 60 and 120 minutes. Reactions were blocked by the addition of EGTA (final concentration 20 mM) and incubation at 65°C for 10 minutes. After phenol-chloroform extraction, DNAs were ethanol-precipitated, resuspended in water and digested for 12 hours with 10 units of HinfI (Thermo Scientific) per μg of DNA. Digested DNA was electrophoresed in 1% agarose gel, denatured and transferred to a nylon membrane (Amersham Hybond-N, GE Healthcare). Membranes were then hybridized with a 32P-α[dCTP]-labeled telomeric probe and exposed to autoradiografic films as previously described [56]. HindIII-digested lambda genomic DNA and GeneRuler 1 kb DNA Ladder (Thermo Scientific) were used as molecular weight markers, the size (Kb) and the positions of the markers are indicated on the left. (TIF) [file pone.0142530.s003.tif]

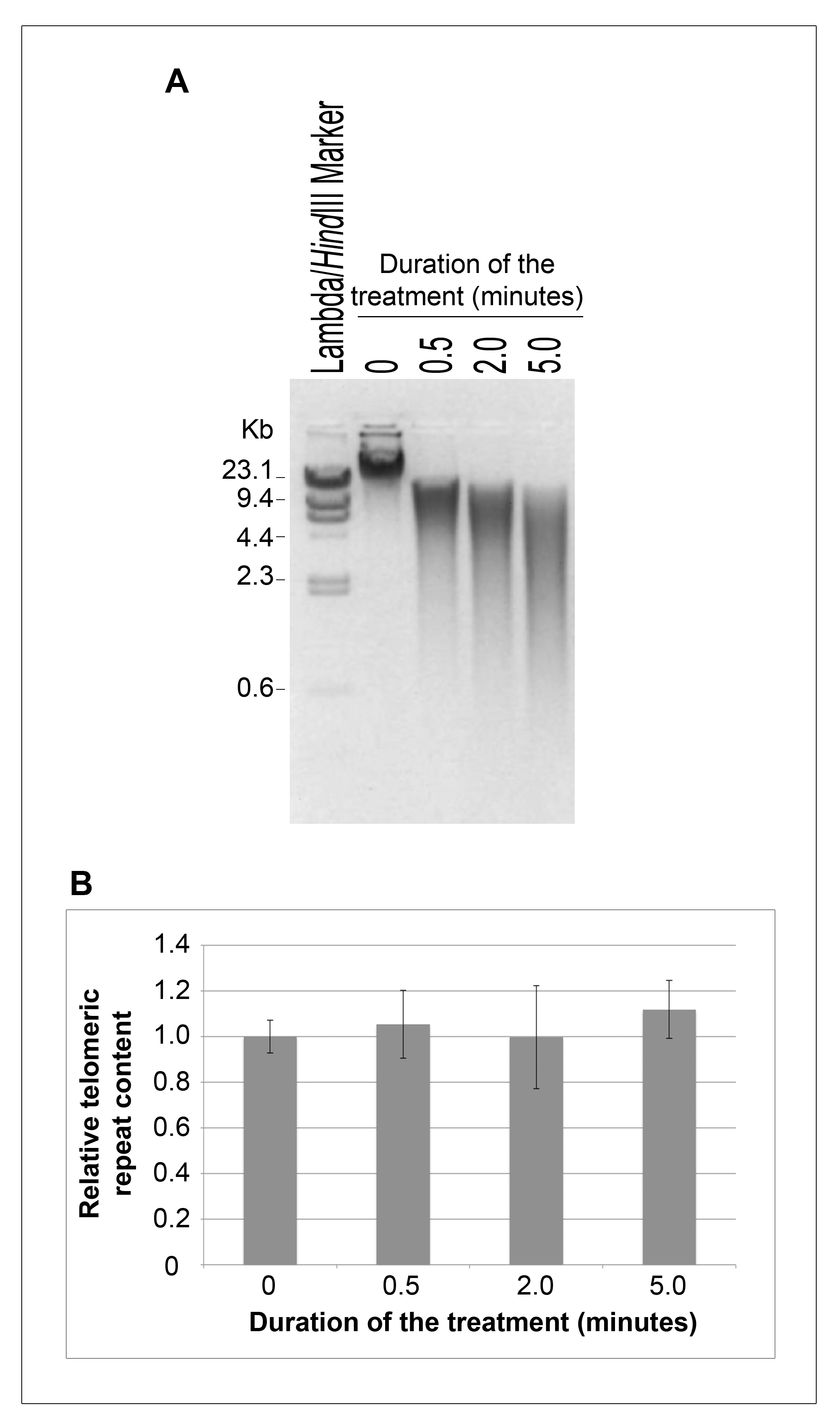

Supplement: S3 Fig — (A) Agarose gel electrophoresis of barn swallow genomic DNA digested with DNase I. (B) Relative telomeric repeat content. Barn swallow genomic DNA was digested with 0.001 units of RNase-free DNase I (Thermo Scientific) per μg of DNA in 1x Reaction Buffer (Thermo Scientific) at 37°C. Aliquots containing 2 μg of DNA were withdrawn after 0, 0.5, 2 and 5 minutes. Digestion was blocked by the addition EDTA to a final concentration of 5 mM and incubation at 65°C for 10 minutes. Degradation was checked by electrophoresis in 1% agarose gel. Digested DNA was ethanol-precipitated and resuspended in 1x nTE. Relative telomeric repeat content was measured by MMQPCR as described in Methods. The relative telomeric repeat content of the untreated sample (0 minutes) was used as reference. (TIF) [file pone.0142530.s004.tif]

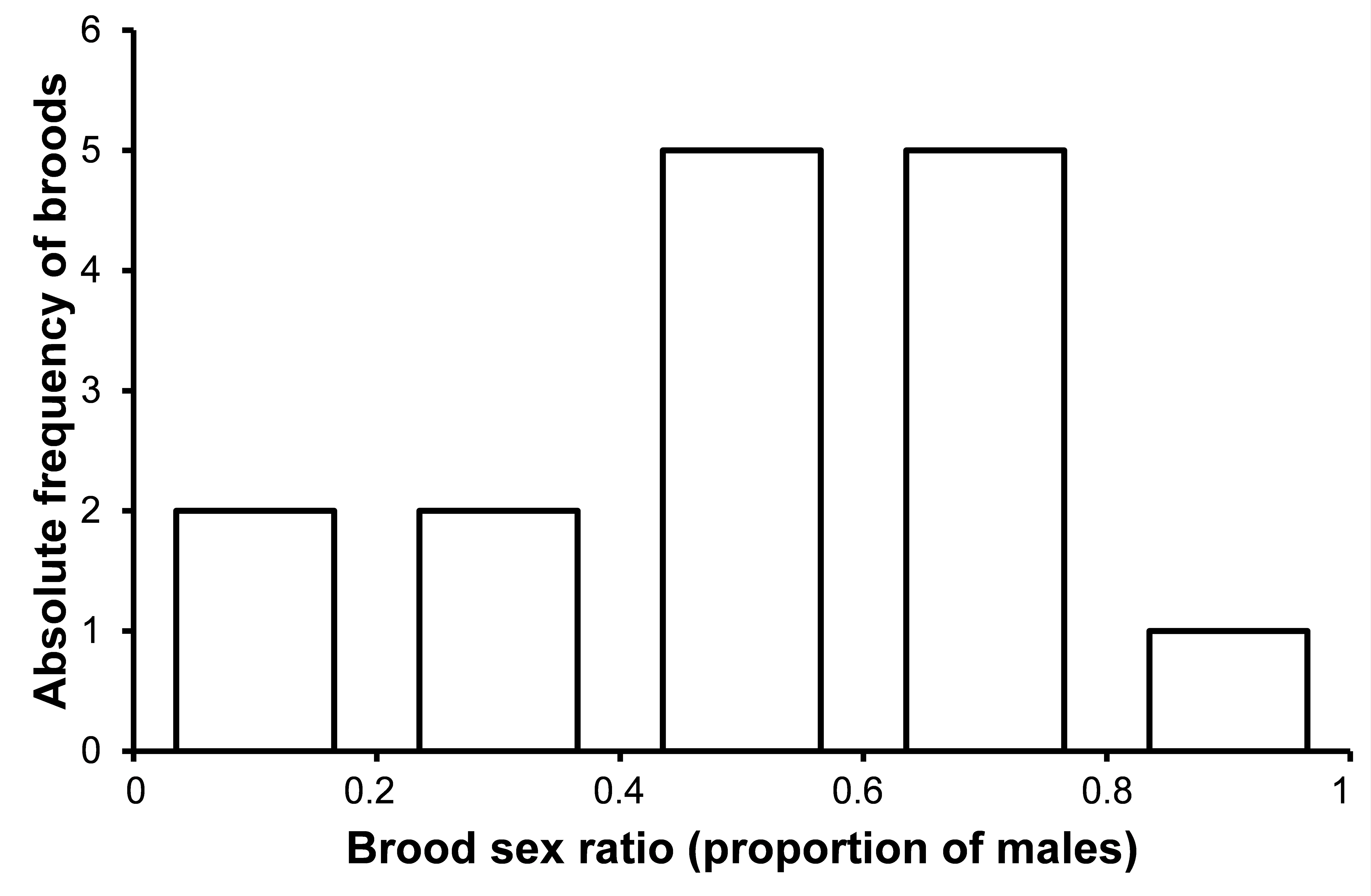

Supplement: S4 Fig — (TIF) [file pone.0142530.s005.tif]

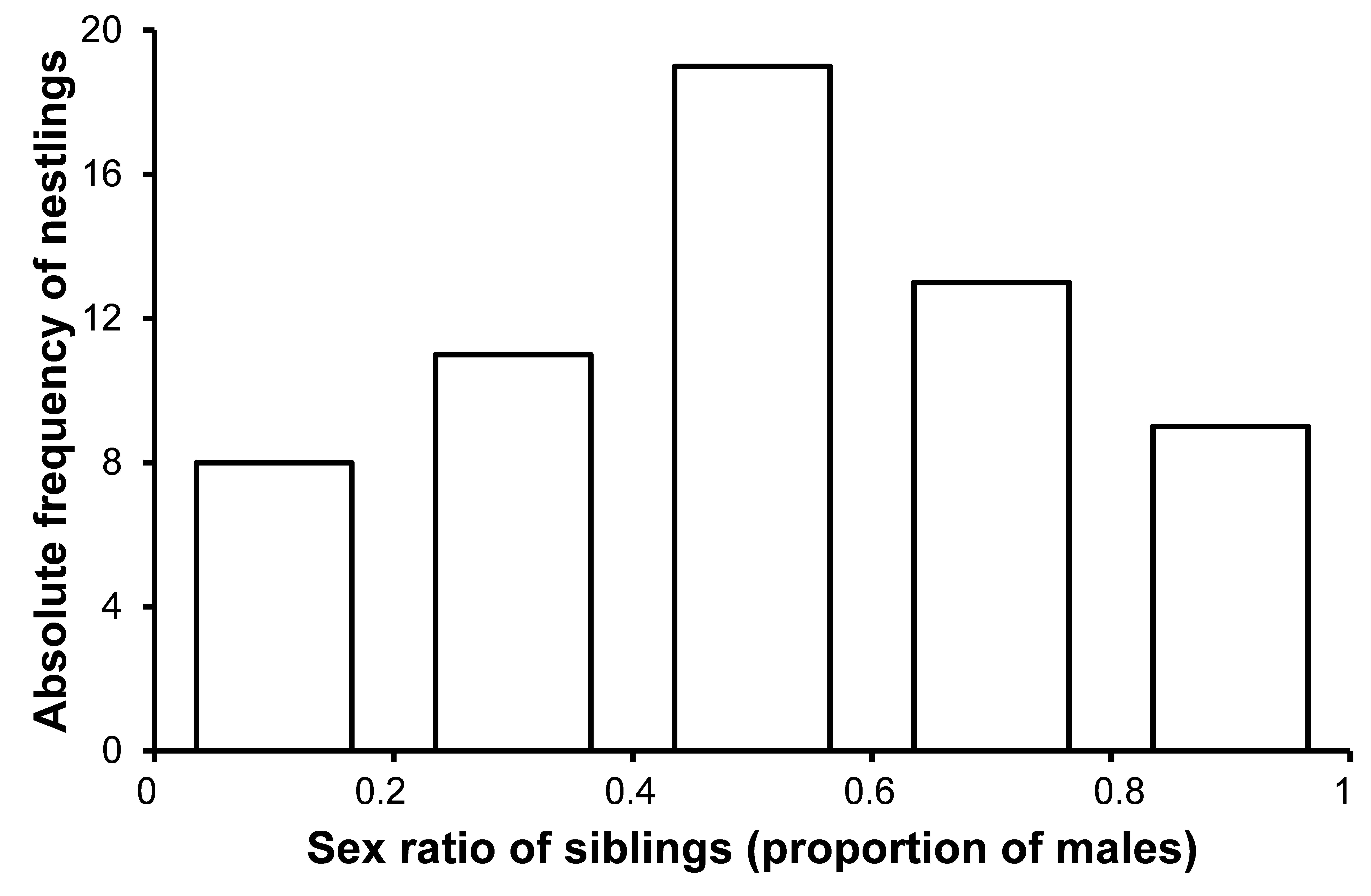

Supplement: S5 Fig — For example, a sex ratio of siblings = 0.5 indicates that half of the siblings of a particular nestling were male and half were female. (TIF) [file pone.0142530.s006.tif]
